# Effect of Pyruvate Decarboxylase Knockout on Product Distribution Using *Pichia pastoris* (*Komagataella phaffii*) Engineered for Lactic Acid Production

**DOI:** 10.3390/bioengineering5010017

**Published:** 2018-02-16

**Authors:** Nadiele T. M. Melo, Kelly C. L. Mulder, André Moraes Nicola, Lucas S. Carvalho, Gisele S. Menino, Eduardo Mulinari, Nádia S. Parachin

**Affiliations:** 1 Grupo de Engenharia Metabólica Aplicada a Bioprocessos, Instituto de Ciências Biológicas, Universidade de Brasília, CEP 70.790-900 Brasília-DF, Brazil; nadytamires@gmail.com (N.T.M.M.); u.lucas@gmail.com (L.S.C.); 2 Pós-Graduação em Ciências Genômicas e Biotecnologia, Universidade Católica de Brasília, CEP 70.790-900 Brasília-DF, Brazil; 3 Integra Bioprocessos e Análises, Campus Universitário Darcy Ribeiro, Edifício CDT, Sala AT-36/37, CEP 70.790-900 Brasília-DF, Brazil; kellylmulder@gmail.com (K.C.L.M.); gisele.sa27@gmail.com (G.S.M.); edumulinari@gmail.com (E.M.); 4 Faculty of Medicine, University of Brasilia, Campus Universitário Darcy Ribeiro, Faculdade de Medicina, sala BC-103, CEP 70.790-900 Brasília-DF, Brazil; andre.nicola@gmail.com

**Keywords:** *Pichia pastoris*, pyruvate decarboxylase, lactic acid, homologous recombination, arabitol, redox metabolism

## Abstract

Lactic acid is the monomer unit of the bioplastic poly-lactic acid (PLA). One candidate organism for lactic acid production is *Pichia pastoris*, a yeast widely used for heterologous protein production. Nevertheless, this yeast has a poor fermentative capability that can be modulated by controlling oxygen levels. In a previous study, lactate dehydrogenase (LDH) activity was introduced into *P. pastoris,* enabling this yeast to produce lactic acid. The present study aimed to increase the flow of pyruvate towards the production of lactic acid in *P. pastoris*. To this end, a strain designated GLp was constructed by inserting the bovine lactic acid dehydrogenase gene (LDHb) concomitantly with the interruption of the gene encoding pyruvate decarboxylase (PDC). Aerobic fermentation, followed by micro-aerophilic culture two-phase fermentations, showed that the GLp strain achieved a lactic acid yield of 0.65 g/g. The distribution of fermentation products demonstrated that the acetate titer was reduced by 20% in the GLp strain with a concomitant increase in arabitol production: arabitol increased from 0.025 g/g to 0.174 g/g when compared to the GS115 strain. Taken together, the results show a significant potential for *P. pastoris* in producing lactic acid. Moreover, for the first time, physiological data regarding co-product formation have indicated the redox balance limitations of this yeast.

## 1. Introduction

Lactic acid has a high commercial value due to its broad application in several areas of industry, such as the automobile, food, pharmaceutical, and textile industries, in addition to the production of biodegradable polymers such as poly-lactic acid (PLA) [1]. The production of lactic acid by fermentation becomes economically feasible when compared to chemical synthesis, as microorganisms can be modified to produce a single isomer. The production of only one isomer facilitates the process of purification and polymerization into poly l-lactic acid (PLLA), which is mainly used in biomedical applications [2]. Moreover, the metabolic conversion of l-lactic acid in humans is much faster when compared to d-lactic acid, thus being preferentially employed in the food and medical sectors [3]. Approximately 82% of the world’s lactic acid production is used by the food industry for microbial fermentation in sauerkraut, yogurts, and butter, among others. Moreover, lactic acid also functions as a pH reducer, solvent, antimicrobial agent, humectant, flavor adjuvant and emulsifier [4]. Due to its various applications, it is estimated that the lactic acid market will be valued at USD $3.82 billion by 2020, which would represent an annual growth rate of 18.6% (https://www.marketsandmarkets.com/Market-Reports/polylacticacid).

Many yeast species have been genetically modified in order to produce lactic acid, including *Saccharomyces cerevisiae* [5,6,7], *Kluyveromyces lactis* [8,9], *Zygosaccharomyces bailii* [10], *Candida* sp. [11,12], and *Pichia* sp. [13]. The yeast *P. pastoris* has been reclassified into the new gender Komagataella, and sub-divided into the three species *K. pastoris*, *K. phaffii* and *K. pseudopastoris* [14]. It has, as its most notable physiological feature, the ability to grow in media containing only methanol as a carbon source [15]. Another advantage is that this yeast grows as fast on crude glycerol as on glucose [16], and can utilize crude glycerol without being inhibited by its impurities [17]. Crude glycerol is the main residue during biodiesel production. For example, Brazil, the second largest biodiesel producer worldwide, reported a production of approximately 4.3 million cubic meters of biodiesel in 2017, which resulted in an estimated 429,129.4 m^3^ of crude glycerol (http://www.anp.gov.br/wwwanp/dados-estatisticos). Thus, the use of glycerol for lactic acid production is advantageous since it adds value to the biodiesel production chain.

The most frequently used and commercially available *K. phaffii* strains are GS115 and X-33, which are derived from the wildtype CBS7435 [18]. The genome of the latter is arranged in four chromosomes, with 5313 open reading frames identified [19]. The GS115 strain is known for its mutation in the enzyme *histidinol dehydrogenase* (HIS4), which makes it auxotrophic for histidine. Another strain derived from CBS7435 is the Ku70 mutant [20]. This harbors the deletion of the gene Ku70, resulting in the absence of a protein involved in the non-homologous end joining repair mechanism. Its deletion is reported to significantly increase the efficiency of homologous recombination and reduce false positives [20].

*P. pastoris* strain GS115 has an annotated gene in its genome that encodes for a putative lactate dehydrogenase (LDH) enzyme (EC 1.1.1.27). However, when growing it on glycerol as a substrate, lactate production is almost absent. In a recent study, this strain was engineered for lactic acid production through the insertion of a LDH activity [21]. Here, for the first time to the authors’ knowledge, deletion of a pyruvate decarboxylase (PDC)-encoding gene has been performed in combination with LDH over-expression, with the aim of funneling further pyruvate to the lactic acid production pathway. Genetically modified *PDC*-knockout strains, as well as GS115, were used in oxygen limited cultivation in order to assess substrate consumption, lactic acid production, and by-product formation. In the *PDC*-deleted strains a 32% and 75% reduction of biomass and acetic acid production was observed, respectively, when compared to the wildtype strain GS115. However, a 2.6-fold increase in arabitol production was observed. In addition, arabitol production increased nearly seven-fold when LDH activity was associated with *PDC* disruption, demonstrating that this alcohol is now the main byproduct of recombinant lactic acid production in the strains of *P. pastoris* tested.

## 2. Materials and Methods

### 2.1. Strains and Plasmids

All of the plasmids and strains used in this study are listed in Table 1. The *Escherichia coli* strains utilized during the cloning steps were grown at 37 °C in Luria broth media (0.5% yeast extract, 1% peptone and 0.5% sodium chloride) and supplemented with ampicillin (100 µg/mL^−1^). The *P. pastoris* strains were grown at 30 °C in YPD (1% yeast extract, 2% peptone and 2% dextrose) and supplemented with Geneticin (G418) (500 µg/mL^−1^) and/or zeocin (100 µg/mL^−1^) when necessary.

### 2.2. Identification of Putative Genes Encoding Pyruvate Decarboxylase

The NCBI platform was used to identify putative genes encoding the PDC enzyme in the genome of the yeast *Komagataella phaffii* GS115. The search for the *P. pastoris* pyruvate decarboxylase gene revealed an ORF annotated as coding for pyruvate decarboxylase XP_002492397, as well as two putative isozymes: XP_002492304 and XP_002492397. Therefore, the reference enzyme sequence chosen for this work was XP_002492397, which refers to the ORF annotated as pyruvate decarboxylase, and previously kinetically characterized in *S. cerevisiae* [22].

### 2.3. Construction of a PDC Knockout Cassette

**Figure 1:**
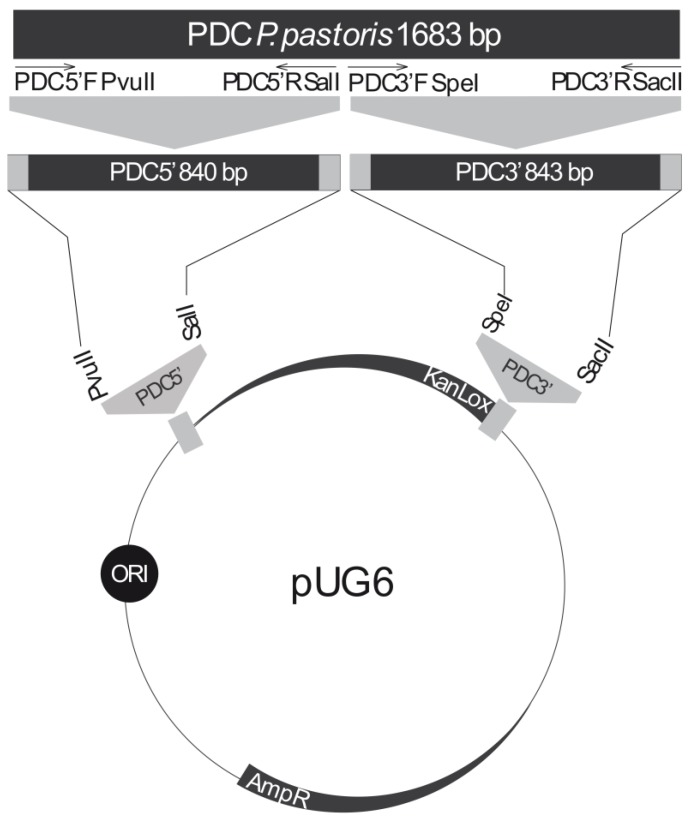
Construction of *PDC* knockout cassette. The entire PDC-encoding gene has 1683 bp. This was divided into two fragments of 840 and 843 bp. Each fragment was cloned into the pUG6 plasmid flanking the Kanamycin resistance cassette with the indicated restriction enzymes. Final cassette has a total of 3317 Kb.

Once the DNA sequence for the PDC enzyme had been obtained, PCR was performed to amplify the complete ORF. For this, the genomic DNA of *P. pastoris* GS115 was used as a template. The primers used were PDC5’F and PDC3’R (Table 2), and the fragment amplified was approximately 1.6 Kb, matching the expected size. The strategy used for construction of the PDC interruption cassette is summarized in Figure 1. For *PDC* gene interruption, the sequence was divided into two fragments: PDC5’ and PDC3’ (840 and 843 base pairs, respectively—see Figure 1). First, the pUG6 plasmid was treated with *Pvu*II and *Sal*I and the PDC5’ fragment was amplified using the primers PDC5’F and PDC5’R (Table 2). The PDC5’ fragment was then cloned into the vector at the upstream region from the kanamycin resistance marker. After confirmation by PCR, the plasmid was treated with the restriction nucleases *Sep*I and *Sac*II. The PDC3’ fragment, amplified by PCR using the primers PDC3’F and PDC3’R (Table 2), was then cloned downstream from the kanamycin resistance marker and its insertion was also confirmed by PCR (data not shown). PCR amplifications were performed in a 20 μL reaction mix containing 2.5 pmol of each primer, 2.5 units Taq DNA polymerase, 0.2 μM of each dNTP, 2.0 µL 10× reaction buffer (10 mM Tris-HCl pH 8.3, 50 mM KCl, 1.5 mM MgCl_2_), and 50 ng of chromosomal DNA. Amplifications were performed with the following conditions: 95 °C for 30’, 95 °C for 15’, 58 °C for 30’, 72 °C for 3 min (30 cycles) and 72 °C for 10 min. This vector was named pUG6-PDCK, and it contained a cassette composed of PDC5’, followed by the kanamycin marker and the PDC3’, a fragment of 3.3 Kb in all.

### 2.4. PDC Knockout in P. pastoris Strains

The *P. pastoris* strains X-33, Ku70, XL and GS115 (Table 1) were transformed with the pUG6-PDCK cassette after linearization with PvuII and SacII. Transformation was carried out by electroporation, following the Easy Select *P. pastoris* Expression Kit (Invitrogen, EUA) protocol, with modifications. Briefly, a single colony was inoculated into 25 mL of YPD medium, and after 24 h at 28 °C and 250 rpm, 1 mL of cells was used to inoculate 100 mL of YPD medium. When the OD_600nm_ reached approximately 1.5 the cells were collected by centrifugation and re-suspended three times with 50 mL of cold and sterile water, followed by one step with 5 mL of cold 1 M sorbitol. All centrifugation steps were performed at 1500× *g* for 5 min at 4 °C. Afterward, the cells were re-suspended in 150 μL of cold 1 M sorbitol and 80 μL of these cells were homogenized with 5–10 μg DNA. This mix was then transferred to a 2 mm electroporation cuvette (Bio-Rad, Berkeley, CA, USA) and incubated on ice for 5 min. An electrical pulse was applied with the following conditions: 1500 V, 400 Ω, and 25 μF. Immediately after the pulse, 1 mL of cold 1 M sorbitol was added and the cells were incubated at 30 °C for 1 h. Finally, the cells were plated in YPD medium supplemented with geneticin (G418) (500 µg/mL^−1^) and incubated at 30 °C for three days. The GS115 strain transformed with the cassette from pUG6-PDCK was then named Gp. The integrative plasmid pGAP-LDH from a previous study [21], harboring the codon-optimized *LDH* encoding the LDH enzyme from *Bos taurus*, was linearized using AvrII and inserted into the Gp strain, resulting in the GLp strain (Table 1).

### 2.5. Ploidy Determination in P. pastoris Strains

Inoculum of 5 mL YPD containing either X-33 or GS115 cells was grown at 30 °C and 200 rpm until the log phase. The cells were then centrifuged at 1500 *g* for 5 min and maintained for 12 h at 4 °C in 10 mL of cold ethanol (70%). After centrifugation for 5 min at 2500 *g*, the cells were washed with 1 mL of 50 mM sodium citrate (pH 7.5). The centrifugation was then repeated and the cells were resuspended in 1 mL of 50 mM sodium citrate (pH 7.5), containing 0.25 g/L RNAse (250 mg/mL). After 1 h at 55 °C, 50 μL of proteinase K (20 mg/mL) was added to the cell suspension. After a further hour at 55 °C, the permeabilized cells were washed, counted and resuspended at a concentration of 10^7^ cells/mL in PBS supplemented with 50 µg/mL propidium iodide. Following 30 min of incubation, the cells were analyzed in a FACS Verse flow cytometer (BD Biosciences) equipped with a 488 nm laser. All samples were collected with the same cytometer settings, and propidium iodide fluorescence was set in linear mode. A forward angle versus side angle scatter area gate was used to remove debris and a forward scatter width versus forward scatter height gate was used for doublet discrimination. The experiment was repeated in triplicate.

### 2.6. LDH Enzyme Activity

Enzyme assays were carried out as described previously, with modifications [23]. Briefly, a primary inoculum culture was prepared in YPD medium, with zeocin (100 μg/mL), and maintained at 30 °C and 180 rpm overnight. Cells were harvested, re-inoculated in a new flask, and grown in a shaker at 30 °C until the exponential phase. After centrifugation, cells were resuspended in Yeast Protein Extraction Reagent (Y-Per, Thermo Scientific, Rockford, IL, USA) for 10 min. The reaction was assembled with 10 µL cellular extract, 8 µL NADH, 800 µL 50 mM phosphate buffer (pH 7), and ultra-pure water for a 1 mL final volume. After 150 s, 40 µL of pyruvate was added and the reaction was completed in 300 s. A unit of enzyme activity was defined as the amount of enzyme necessary to oxidize 1 μmol of NADH per minute. Protein concentration of cell extracts was determined using the Coomassie protein assay reagent (Pierce, Rockford, IL, USA) according to the manufacturer’s instructions. BSA in known concentrations was used to construct the standard curve. Enzyme assays were carried out in three biological replicates.

### 2.7. Fermentation Parameters

For cultivations in the bioreactors, a defined medium was utilized as previously described [24], with modifications. The composition of the medium (per liter) was: 1.8 C_6_H_8_O_7_, 0.02 g CaCl_2_·2H_2_O, 12.6 g (NH_4_)2HPO_4_, 0.5 g MgSO_4_·7H_2_O, 0.9 g KCl and 4.35 mL PTM1 trace salts stock solution. pH was adjusted to 5.0 with 25% HCl. PTM1 trace salts stock solution (per liter) was composed of: 6 g CuSO_4_ 5H_2_O, 0.08 g NaI, 3 g MnSO_4_·H_2_O, 0.2 g Na_2_MoO_4_ 2H_2_O, 0.02 g H_3_BO_3_, 0.5 g CoCl_2_, 20 g ZnCl_2_, 14.3 g FeSO_4_, 0.4 g biotin and 5 mL H_2_SO_4_ (95–98%). 0.04 g/L histidine was supplemented for the GS115 strain.

A 100 mL pre-culture was prepared with 20 g/L glycerol, and was grown for approximately 48 h at 30 °C and 200 rpm in a 1 L bioreactor (Infors HT., Bottmingen, Switzerland). Cultivations in the bioreactors were performed with 500 mL medium at an initial OD_600nm_ of 2, and with initial glycerol concentration of 80 g/L. The batch phase was performed under the following conditions: 30 °C, 500 rpm, dissolved oxygen at 30%, and pH 5.0 controlled with 5 M NH_4_. Feeding started after glycerol depletion by supplementing the culture with 40 g/L glycerol in a single pulse. When the pH went above 5.0, dissolved oxygen was kept at 3%. Samples were collected every 90 min and centrifuged at 12,000 g for 2 min. The supernatant was stored at −20 °C for HPLC analysis.

### 2.8. Substrate Consumption and Cellular Products Quantification

Glycerol, lactic acid, acetic acid, ethanol and arabitol were quantified using High-performance Liquid Chromatograph (HPLC) (Shimadzu, Kyoto, Japan) equipped with UV (210-nm) and refractive index detectors as previously described [25]. A pre-column Guard Column SCR (H) (50 mm × 4 mm id) with stationary phase sulfonated styrene-divinylbenzene copolymer resin was used. The chromatography flow rate was 0.6 mL/min and an injection volume of 20 µL, using a Shim-pack SCR-101H (Shimadzu) (300 mm × 7.9 mm id) column equilibrated at 60 °C with 5 mM H_2_SO_4_ as the mobile phase. For biomass determination, samples collected for OD_600nm_ measurement were dried and then weighed for analysis of biomass dry cell weight (DCW). A calibration curve (1 unit of OD_600nm_ corresponded to 0.31 g DCW/L) was used to convert OD_600nm_ to DCW (g/L).

## 3. Results and Discussion

### 3.1. Construction of pUG6-PDCK and PDC Knockout P. pastoris Strains

Pyruvate Decarboxylase activity has been deleted in other yeast species to reduce by-product formation such as acetate and ethanol as well as concomitant increases in lactic acid production [5,6,26]. Therefore, in order to verify whether lactic acid production could also be increased in *P. pastoris*, the PDC gene was interrupted. To that end, a knockout cassette with a kanamycin resistance marker was constructed (Figure 1) and used for yeast transformation.

**Figure 2:**
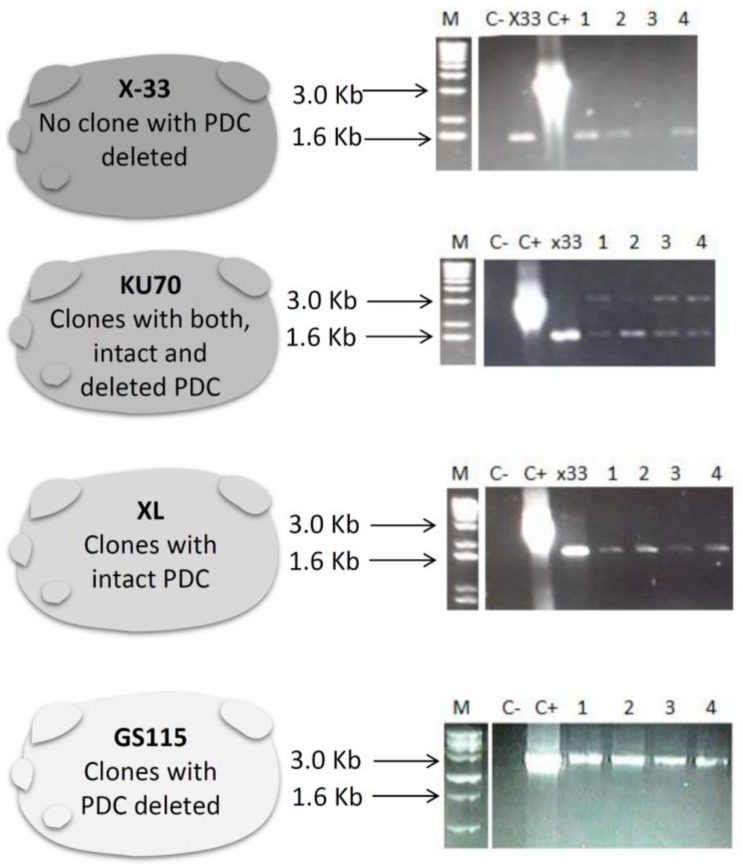
The four *P. pastoris* strains used for the insertion of the *PDC* knockout cassette with their respective results among all screened clones. The PCR results using the primers PDC5’F and PDC3’R are shown after electrophoresis on a 0.8% agarose gel. Kb: kilobases; M: molecular marker; C−: negative control (no DNA template in the PCR reaction); C+: positive control (Plasmid pUG6-PDCK as template in the PCR reaction), X-33-*Pichia pastoris* wildtype strain where *PDC* is not deleted. Numbers 1–4 are samples of different clones that were evaluated for deletion of *PDC*.

After transformation into the *P. pastoris* X-33 strain, 300 colonies were screened for the *PDC*-knockout cassette using PCR amplification, without success. The amplification of a fragment size of approximately 3.3 Kb was expected, indicating the insertion of the cassette. However, only the fragment of 1.6 Kb was detected, showing amplification of only the intact *PDC* gene (Figure 2 upper panel).

Therefore, to improve the efficiency of homologous recombination, the Ku70 strain was utilized. This strain has been previously described as having an increased frequency of homologous recombination [20]. After yeast transformation, all the selected clones presented amplification of both intact *PDC* (1.6 Kb) and *PDC*-knockout (3.3 Kb) (KU070 results in Figure 2). All clones were then re-plated on YPD supplemented with geneticin (G418) (500 µg/mL^−1^), 5 times. After the final plating, isolated colonies had their genomic DNA extracted and used as template for PCR reactions. Of these, all the clones presented only the 1.6 Kb fragments, which indicated the presence of intact *PDC* (data not shown).

The deletion of the *PDC* gene has been shown to reduce or impair growth in other yeast species [8,27], which may explain why no single Ku70 colony with interrupted *PDC* could be isolated. Therefore, the *P. pastoris* XL strain that has lactate dehydrogenase activity [21] was tested. The hypothesis was that the presence of a novel route for NAD regeneration, which was not respiratory, could reestablish growth in PDC-defective strains. Nevertheless, after screening approximately 200 colonies that were resistant to Geneticin (G418), all clones had amplification profiles matching intact *PDC* (XL results in Figure 2). To understand why antibiotic resistant colonies did not have *PDC* knockout, PCR using the specific primers KanF and KanR (Table 2) as a Geneticin marker were utilized. In all tested colonies, the amplification of uninterrupted *PDC* genes and the Geneticin-resistant marker were observed (data not shown). Hence, the resistance marker was integrated into *P. pastoris* genome by non-homologous integration.

Lastly, transformation of the *P. pastoris* GS115 strain was performed. This strain has the HIS4 enzyme gene deleted, and is considered the most popular strain for production of heterologous proteins [28]. Since it has a well-established deletion in its genome, the knockout of *his4*, it was hypothesized that it could facilitate a further insertion. After GS115 transformation, colonies selected in the presence of geneticin (G418) had their genomic DNA extracted. As can be seen in the lower panel of Figure 2, evaluation of all clones resulted in the amplification of a 3.3 Kb fragment that indicated the insertion of the *PDC* knockout cassette into the yeast chromosome (Table 2).

### 3.2. Determination of Ploidy in Different P. pastoris Strains

In order to understand why *PDC* knockout was easily performed in GS115 and could not be achieved in a single colony for X-33, Ku70 and XL, flow cytometer experiments were performed to determine the total amount of DNA for each strain. This technique has been previously utilized for separating metabolically-defective cells during fed-batch cultures of recombinant X-33 producing both trypsinogen and horseradish peroxidase [29]. The X-33 strain has been described as wildtype and there are no reports stating whether it is haploid [30]. Ku70 and XL, being derivatives of X33, should have, in theory, the same DNA content. Finally, the GS115 strain has been described as haploid and has been generated by random mutagenesis from the parental strain NRRL Y-11430 [18,31]. Flow cytometer experiments showed that X33 and GS115 have the same DNA content, thus not explaining why integration occurred in the PDC fragment in GS115 and not in X-33 (Supplementary Material S1). As such, a possible explanation for obtaining amplification of the deletion cassette and intact PDC in Ku70 strains would be the integration of the entire deletion cassette in another region of the genome.

However, this does not explain the successful results obtained when *PDC* knockout was attempted in these strains (GS115). For the strains where *PDC* intact and knockout profile could be detected within the same colony, serial dilution was carried out unsuccessfully in order to obtain a pure profile (data not shown). Therefore, it has been hypothesized that as happens in other yeasts such as *S. cerevisiae* [5,6] and *K. lactis* [8,9], *PDC* knockout reduces biomass which makes its selection difficult when performing serial dilution. Later in this study, the fermentation results corroborated this hypothesis where a decrease of 35% of biomass could be seen in Gp when compared to GS115 (Table 3).

### 3.3. Product Distribution in P. pastoris Strains during Bioreactor Cultivation

After confirming the deletion of *PDC* in GS115 generating the GP strain (Table 1), the *LDHb* gene was inserted into that strain to produce lactic acid, and this was designated the GLp strain (Table 1). Subsequently, LDH activities were measured in both the XL and GLp strains (Supplementary Information S2). As can be seen, GLp had about 40% lower LDH activity when compared to GL, most probably due to a difference in copy integration of the LDH-encoding gene. Nevertheless, since this was the highest activity achieved in a strain where PDC was interrupted, the experiments were conducted using this strain.

**Figure 3:**
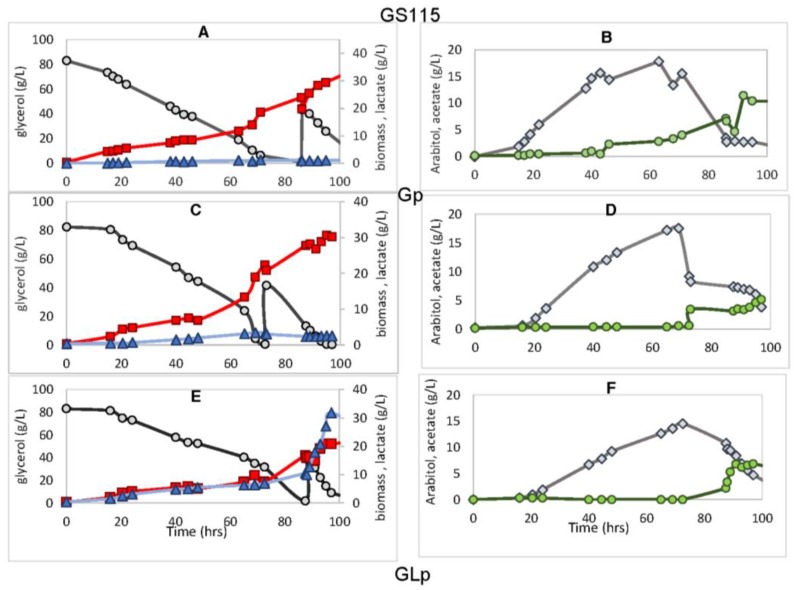
Fermentation profile of the strains GS115 (**A**,**B**), Gp (**C**,**D**), and GLp (**E**,**F**) showing consumption of glycerol (gray circle) and the production of biomass (red square), lactate (blue triangle), arabitol (gray diamond) and acetate (green circle). 4% glycerol was added at 70–80 fermentation hours when oxygen was limited. Experiments were performed in biological triplicates, and the figure shows a typical fermentation profile.

The impact of both *PDC* gene knockout and insertion of the bovine lactate dehydrogenase gene on the production of l-lactic acid in *P. pastoris*, as well as the evaluation of by-products such as ethanol, acetate and arabitol, were investigated (Figure 3 and Table 3). The cultivations in the bioreactors were divided into two phases (Figure 3). In the first, termed the aerobic phase, the dissolved oxygen was maintained at 30%, and 8% glycerol was fed to the strains. In the second, which was initiated by pH level alteration to >5.0, the dissolved oxygen was limited to 3%, and this was therefore named the restricted aerobic phase, where 40% glycerol was added in a single pulse.

The Gp strain harboring *PDC* knockout reduced acetate formation by approximately 20-fold and 4-fold in the aerobic and restricted aerobic phases, respectively, when compared with GS115 (Table 3). On the other hand, it can be observed that the production of arabitol was approximately 1.5-fold and 3.0-fold higher than GS115 in the same phases, respectively (Figure 3 and Table 3).

Regarding biomass formation, PDC deletion did not reduce biomass formation aerobically. Nevertheless, for the GLp strain that had both *PDC* deleted and insertion of LDH activity, the biomass yield was reduced by approximately 30% and 10% in the aerobic and restricted aerobic phases, respectively, when compared to the Gp strain harboring only *PDC* knockout (Table 3). Nevertheless, when aerobic and oxygen-limited conditions are compared within the same strain, biomass yields are slightly higher for the latter, even though the specific growth rate is lower (Table 3). Since this is not a fermentative yeast even under oxygen-limiting conditions, the main yeast product is still biomass. Altogether these results show that under aerobic conditions the main yeast products are biomass and arabitol for all strains independently of the inserted genetic modification. Nevertheless, under oxygen-limited conditions, the lactate production was favored over biomass, where the lactic acid yield was approximately five times higher than at the aerobic phase.

**Figure 4:**
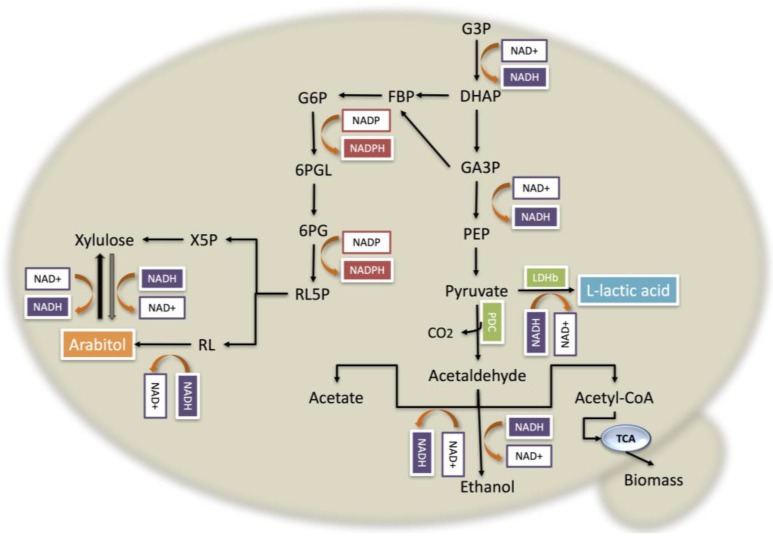
Glycerol metabolism of *P. pastoris* GLp strain showing the main pathways involved in NADH oxidation. G3P: Glycerol-3-phosphate; DHAP: Dihydroxyacetone; GA3P: glyceraldehyde-3-phosphate; PEP: phosphoenolpyruvate; LHHb: bovine l-lactic acid dehydrogenase; PDC: pyruvate decarboxylase; FBP: fructose-biphosphate; G6P: glucose-6-phosphate; 6PGL: phosphogluconolactone; 6PG: phosphogluconate; TCA: tricarboxylic acid; NAD: nicotinamide adenine dinucleotide; NADH: reduced NAD.

However, different than what was observed from the comparison between GS115 and Gp, GLp led to an acetate yield increase of 3-fold for the restricted aerobic phase (Table 3). Although the production of arabitol followed the same pattern, the yield increase was lower from Gp to GLp (1.2-fold and 2.5-fold for aerobic and restricted aerobic phases, respectively), than from GS115 to Gp. Moreover, under oxygen-limited conditions, GLp presented an arabitol yield decrease of approximately 45%, reaching a specific productivity rate of approximately 3.5-fold lower than that observed in the first phase (Table 3). This suggests that during the aerobic phase, excess NADH may be oxidized via arabitol production (Figure 4). On the other hand, under oxygen-limited conditions this was minimized by the addition of the LDH activity, which is also a pathway that leads to NADH oxidation. 

When compared with a previous study, where a putative lactate transporter from *P. pastoris* was inserted into the GS115 strain harboring the *LDHb* gene [21], the GLp strain proved to be a more efficient biocatalyst, converting substrate into l-lactic acid faster than GLS: qlac = 0.146 h^−1^; GLp: qlac = 0.05 h^−1^, while producing the same amount of biomass (GLS: qx = 0.014 g/g/h; GLp: qx = 0.015 g/g/h) (Table 3) [21]. In fact, the yields for GLS and GLp are 0.673 g/g and 0.646 g/g respectively. Although very similar, the GLS strain is much slower at reducing, requiring about 48 h more to achieve the same lactic acid yield.

### 3.4. Effect of PDC Interruption on l-Lactic Acid Production

*P. pastoris* strains engineered for production of lactic acid have not yet reached the maximum theoretical yield [21]. In fact, only a few yeast species have been modified to increase lactic acid yield, including *K. lactis* and *S. cerevisiae* [32,33]. *K. lactis* has only one *PDC* gene, which is non-essential for its survival when grown on glucose [26]. Suppression of this gene together with knockout of the pyruvate dehydrogenase E1 alpha sub-unit gene increases lactic acid production, reaching a yield of 0.85 g/g [8].

*S. cerevisiae* is the most widely studied yeast for production of lactic acid, mainly due to its fermentative metabolism [8,33,34,35]. *S. cerevisiae* has two active structural *PDC* genes, *PDC1* and *PDC5*, and a third inactive gene named *PDC6* [27]. It has been previously reported that double deletion of *PDC1* and *PDC5* led to efficient production of l-lactic acid (82 g/L), however, cell growth was also reduced in the knockout strain [7]. Among the *PDC* genes, *PDC1* has been shown to have the greatest effect on cell growth and suppression of ethanol production (yields from 0.35 g/g to 0.20 g/g), concomitant with an improvement in lactate yield from glucose (from 0.155 g/g to 0.20 g/g) [36]. Another study has shown that double disruption of the *PDC1* and alcohol dehydrogenase genes results in an almost two-fold increase in l-lactic acid production, reaching a yield of 0.75 g/g in glucose [5]. However, it has been recently reported that while *LDH A* from the fungus *Rhizopus oryzae* has been used instead of the bovine *LDH* gene, no gene deletions are required to reach yields of up to 0.69 g/g of lactic acid using *S. cerevisiae* when xylose is used instead of glucose [37]. This latter study showed that when culturing the yeast on xylose under oxygen-limited conditions, the major fermentation product was lactic acid, showing an increase of 3-fold while ethanol production decreased 30-fold [37].

*PDC* knockout was shown to eliminate acetate production and thus improve lactic acid formation in other yeasts [8,26]. In this study, higher lactic acid titers were also achieved using the strain with interrupted *PDC*. Nevertheless, although acetic acid was reduced, it can be seen that it was not completely eliminated. Examination of the *P. pastoris* genome annotation revealed that this yeast has one gene annotated as *PDC* (XP_002492397) and two other putative PDC enzymes. Since we deleted only XP_002492397, our hypothesis is that the other isoforms are still converting pyruvate to acetaldehyde.

### 3.5. The Redox Mechanism When PDC Is Interrupted

The insertion of the *PDC* knockout cassette into the GS115 strain led to a 1.6-fold increase in arabitol production (Table 3). Arabitol belongs to the pentitol family and is used in the food, health and chemical industries [38]. It has been demonstrated that several yeasts, mainly from the genera *Debaryomyces*, *Geotrichum*, *Metchnikowia*, *Candida* and *Dipodascus*, are able to produce significant amounts of arabitol from glycerol (up to 41.7 g/L) [39]. The *P. pastoris* GS115 strain has been previously shown to produce d-arabitol from glucose [40].

In yeast cells, arabitol synthesis from glycerol is expected to follow similar routes to those leading from glucose [38]. According to the genome annotation, after glycerol is assimilated by the cells, it follows two steps of phosphorylation and is converted into pyruvate, producing two NADH molecules (Figure 4). This latter compound may then follow one of two pathways, one being the conversion to glyceraldehyde-3–phosphate (GA3P) by the enzyme triose phosphate isomerase, with subsequent conversion to pyruvate, which is in turn converted into l-lactic acid by the NADH-dependent bovine lactate dehydrogenase, resulting in a NADH surplus per mol of consumed glycerol under oxygen-limited conditions.

In the GLp strain, since the *PDC* gene is interrupted, NADH cannot be regenerated under oxygen-limited conditions by the formation of ethanol. This unbalanced co-factor concentration would then drive DHAP into arabitol formation by reduction of either ribulose or xylulose through reoxidation of NADH, which then reestablishes the redox balance of the yeast (Figure 4). Indeed it has been previously reported for *P. pastoris* that the effect of reduced oxygen supply from 21% to 8% in the core metabolism led to an increase in arabitol yield of 220%, reflecting adaptation from a respiratory to a respiratory-fermentative metabolism [41].

## 4. Conclusions

In this study, the disruption of *P. pastoris* genes required the analysis of many clones, since homologous recombination was not highly efficient even when performed with more than 500 bp of homology. Although different strain backgrounds were tested for deletion of the *PDC* gene by homologous recombination, a positive result was only achieved for GS115. Fermentation experiments showed that during the oxygen-limited phase the GLp strain achieved a lactic acid yield of 0.65 g/g. In this strain, acetate formation was reduced by 20% but with a concomitant 7-fold increase in arabitol production when compared to wildtype strain GS115. This may be explained as a route for NADH regeneration under oxygen-limited conditions.

## Figures and Tables

**Table 1:** Plasmids and strains used and developed in this work.

Plasmids	Relevant Genotype	Ref.
pUG6	loxP-PTEF-KanMX-TTEF-loxP	Life Technologies
pGAP-LDH	LDH^+^. *Bos taurus* gene encoding the LDH enzyme	[21]
pUG6-PDC	loxP-PTEF-KanMX-TTEF-loxP+PDC-	This work
Strains	Relevant Genotype	Ref.
DH5α™	F-Φ80*lac*ZΔM15 Δ(*lac*ZYA-*arg*F) U169 *rec*A1 *end*A1 *hsd*R17 (rK–, mK+) *pho*A *sup*E44 λ-*thi*1 *gyr*A96 *rel*A1	Life Technologies
X-33	Wildtype	Invitrogen
CBS7435 ku70	Δ*ku70*	[20]
GS115	Δ*his4*	[18]
XL	X-33 + pGAP-LDHBos taurus	[21]
GLp	GS115: Δ*pdc* + pGAP-LDHBos taurus	This work
Gp	GS115:Δ*pdc*	This work

**Table 2:** Primers used in this study. Sequences of restriction enzymes are highlighted in bold.

Primer	Sequence 5’-3’	Endonuclease
*PDC5’F*	**CAGCTG**ATGGCTGAAATAACACTAGGAACT	*Pvu*II
*PDC5’R*	**GTCGAC**ATCAGCCTTCTCCACGAACT	*Sal*I
*PDC3’F*	**ACTAGT**CTTGTCATCTCTGTTGGTGC	*Spe*I
*PDC3’R*	**CCGCGG**TTAAGCTGCGTTGGTCTTGG	*Sac*II
*KanF*	AGCTTGCCTCGTCCCC	
*KanR*	TCGACACTGGATGGCG	

**Table 3:** Kinetic parameters during fermentation experiments at aerobic and oxygen limited phases. Y: yield, s: substrate, x: biomass, lac: lactate, ac: acetate, ara: arabitol, Y: g/g, q: g/g/h, r: g/L/h. Experiments were performed in biological triplicates. Yields during the oxygen limited phase were calculated upon 40% glycerol feeding at the end of the aerobic phase.

Strain	Y_x/s_	Y_lac/s_	Y_lac/x_	Y_ac/s_	Y_ara/s_	µ	q_lac_	q_ac_	q_ara_	
GS115	0.197 ± 0.025	0.015 ± 0.002	0.075 ± 0.002	0.062 ± 0.001	0.170 ± 0.064	0.023 ± 0.001	0.002 ± 0.001	0.007 ± 0.001	0.021 ± 0.011	**Aerobic**
Gp	0.204 ± 0.014	0.045 ± 0.001	0.219 ± 0.012	0.003 ± 0.001	0.247 ± 0.028	0.028 ± 0.005	0.006 ± 0.001	0.000 ± 0.000	0.033 ± 0.009	
GLp	0.138 ± 0.018	0.110 ± 0.003	0.807 ± 0.126	0.008 ± 0.001	0.308 ± 0.003	0.021 ± 0.003	0.017 ± 0.000	0.001 ± 0.000	0.047 ± 0.000	
GS115	0.319 ± 0.021	0.007 ± 0.001	0.022 ± 0.002	0.102 ± 0.000	0.025 ± 0.016	0.015 ± 0.001	0.000 ± 0.000	0.005 ± 0.001	0.002 ± 0.001	**Oxygen Limited**
Gp	0.217 ± 0.014	0.013 ± 0.002	0.058 ± 0.014	0.026 ± 0.011	0.067 ± 0.023	0.017 ± 0.001	0.001 ± 0.000	0.002 ± 0.001	0.006 ± 0.002	
GLp	0.194 ± 0.012	0.646 ± 0.054	3.34 ± 0.072	0.081 ± 0.015	0.174 ± 0.038	0.015 ± 0.003	0.050 ± 0.009	0.006 ± 0.002	0.014 ± 0.007	

## Supplementary Materials

The following are available online at http://www.mdpi.com/2306-5354/5/1/17/s1, Supplementary Figure S1: Permeabilized cells from the P. pastoris KU70 (green), GS115 (orange) and X-33 (Blue) strains were stained with propidium iodide and analyzed by flow cytometry. The histogram distribution of G1 and G2 peaks show that the cells have similar DNA content. The strain X-33 unstained with propidium iodide is shown in red; Supplementary Figure S2. Specific activities for lactate dehydrogenase measured in XL and GLP strains. Activities were measured in biological triplicates.

Supplementary File 1
