# Supplementary material for: Effect of Pyruvate Decarboxylase Knockout on Product Distribution Using Pichia pastoris (Komagataella phaffii) Engineered for Lactic Acid Production
Source: Bioengineering (Basel). 2018 Feb 16;5(1):17. doi: 10.3390/bioengineering5010017 (PMC5874883; doi:10.3390/bioengineering5010017)

**Bioengineering**

**Effect of pyruvate decarboxylase knock-out in product distribution using Pichia pastoris (Komagataella phaffii) engineered for lactic acid production**

Nadiele T. M. Melo^ad#^, Kelly C. L. Mulder^b#^, André Moraes Nicola^c^, Lucas S. Carvalho^a,b^, Gisele S. Menino^b^, Eduardo Mulinari^b^, Nádia S. Parachin^a*^

^a^ Grupo de Engenharia Metabólica Aplicada a Bioprocessos. Instituto de Ciências Biológicas. Universidade de Brasília. CEP 70.790-900 Brasília-DF. Brazil.

^b^ Integra Bioprocessos e Análises. *Campus* Universitário Darcy Ribeiro. Edifício CDT. Sala AT-36/37. CEP 70.904-970 Brasília-DF. Brazil.

^c^ Faculty of Medicine, University of Brasilia. Campus Universitário Darcy Ribeiro, Faculdade de Medicina, sala BC-103. Brasília-DF, 70910-900.

^d^ Pós-Graduação em Ciências Genômicas e Biotecnologia, Universidade Católica de Brasília, Brasília-DF, Brazil.

^#^ These authors contributed equally to this work

**Corresponding author: Grupo de Engenharia Metabólica Aplicada a Bioprocessos. Instituto de Ciências Biológicas. Universidade de Brasília. CEP 70.790-900 Brasília-DF. Brazil. E-mail: nadiasp@unb.br.*

**Supplementary Figure S1.** Permeabilized cells from the *P. pastoris* KU70 (green), GS115 (orange) and X-33 (Blue) strains were stained with propidium iodide and analyzed by flow cytometry. The histogram distribution of G1 and G2 peaks show that the cells have similar DNA content. The strain X-33 unstained with propidium iodide is shown in red.


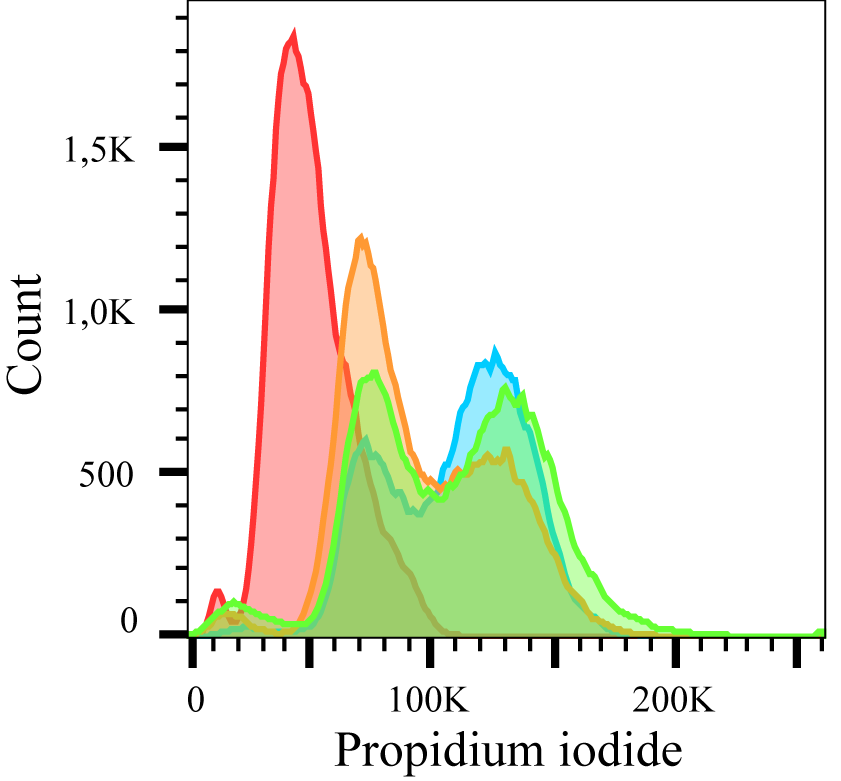


**Supplementary Figure S2.** Specific activities for lactate dehydrogenase measured in XL and GLP strains. Activities were measured in biological triplicates.


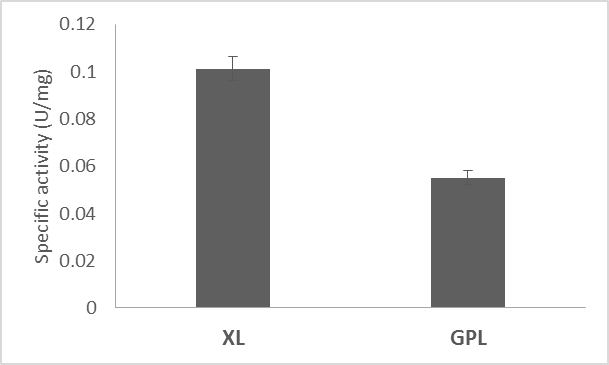

Supplement: Supplementary File 1 [file bioengineering-05-00017-s001.docx]
